# State-of-the-Art of Polymer/Fullerene C_60_ Nanocomposite Membranes for Water Treatment: Conceptions, Structural Diversity and Topographies

**DOI:** 10.3390/membranes13010027

**Published:** 2022-12-25

**Authors:** Ayesha Kausar, Ishaq Ahmad, Malik Maaza, M. H. Eisa

**Affiliations:** 1 NPU-NCP Joint International Research Center on Advanced Nanomaterials and Defects Engineering, Northwestern Polytechnical University, Xi’an 710072, China; 2 UNESCO-UNISA Africa Chair in Nanosciences/Nanotechnology, iThemba LABS, Somerset West 7129, South Africa; 3 NPU-NCP Joint International Research Center on Advanced Nanomaterials and Defects Engineering, National Centre for Physics, Islamabad 44000, Pakistan; 4 Department of Physics, College of Science, Imam Mohammad Ibn Saud Islamic University (IMSIU), Riyadh 13318, Saudi Arabia

**Keywords:** polymer, fullerene C_60_, nanocomposite, membrane, nanofiltration, permeability, selectivity, salt rejection, water treatment

## Abstract

To secure existing water resources is one of the imposing challenges to attain sustainability and ecofriendly world. Subsequently, several advanced technologies have been developed for water treatment. The most successful methodology considered so far is the development of water filtration membranes for desalination, ion permeation, and microbes handling. Various types of membranes have been industrialized including nanofiltration, microfiltration, reverse osmosis, and ultrafiltration membranes. Among polymeric nanocomposites, nanocarbon (fullerene, graphene, and carbon nanotubes)-reinforced nanomaterials have gained research attention owing to notable properties/applications. Here, fullerene has gained important stance amid carbonaceous nanofillers due to zero dimensionality, high surface areas, and exceptional physical properties such as optical, electrical, thermal, mechanical, and other characteristics. Accordingly, a very important application of polymer/fullerene C_60_ nanocomposites has been observed in the membrane sector. This review is basically focused on talented applications of polymer/fullerene nanocomposite membranes in water treatment. The polymer/fullerene nanostructures bring about numerous revolutions in the field of high-performance membranes because of better permeation, water flux, selectivity, and separation performance. The purpose of this pioneering review is to highlight and summarize current advances in the field of water purification/treatment using polymer and fullerene-based nanocomposite membranes. Particular emphasis is placed on the development of fullerene embedded into a variety of polymer membranes (Nafion, polysulfone, polyamide, polystyrene, etc.) and effects on the enhanced properties and performance of the resulting water treatment membranes. Polymer/fullerene nanocomposite membranes have been developed using solution casting, phase inversion, electrospinning, solid phase synthesis, and other facile methods. The structural diversity of polymer/fullerene nanocomposites facilitates membrane separation processes, especially for valuable or toxic metal ions, salts, and microorganisms. Current challenges and opportunities for future research have also been discussed. Future research on these innovative membrane materials may overwhelm design and performance-related challenging factors.

## 1. Introduction

Clean water is vivacious for human beings and the ecological world environment [1,2]. To achieve unsoiled water, implementation of water treatment or purification technologies have been focused on. As the most successful method, the concentrated fabrication and examination of high-performance membrane technologies has been foreseen. Miscellaneous membrane-dependent technologies, therefore, have been applied for water decontamination [3,4]. Usually, membrane technologies have been claimed for having low cost, fine workability, efficiency, low energy ingesting, and scale-up viabilities [5]. Pressure-driven membranes processes have been applied in conventional membrane filtration [6]. Polymeric membranes have been identified for the high permeability, the stability, the selectivity, the salt rejection, and the low working pressure. Polymeric membranes have been developed using various polymers as polystyrene, polyethylene, polyamide, polyacrylonitrile, poly(vinylidene fluoride), poly(vinyl alcohol), and polyaniline [7,8,9]. Common processes used for the formation of filtration membranes include phase inversion, interfacial polymerization, solution casting, sol-gel processes, and electrospinning [10]. Polymeric membranes have been characterized for the morphology, crystallinity, hydrophilicity, surface roughness, permeation, flux, salt rejection, and antifouling performances. However, the foremost downsides of neat polymeric membranes include the irregularly porous structure, hydrophobicity, membranes fouling, and pore clogging due to effluents [11]. Therefore, the membrane technology has shifted towards the use of nanocomposite membranes instead of neat polymeric membranes [12,13]. For the formation of polymeric nanocomposite membranes, inorganic and organic nanoparticles have been considered [14]. Inorganic nanoparticles such as metal or metal oxides such as Au, Ag, Fe, SiO_2_, Al_2_O_3_, zeolite, and polyhedral oligomeric silsesquioxanes have been filled to form innovative polymeric nanocomposite membranes [15]. Nanocarbon nanomaterials such as fullerene, carbon nanotubes, graphene, nanodiamonds, and carbon nanofiber have also been used to form nanocomposite membranes [16,17,18]. Among various types of nanofiller, carbonaceous nanofillers have revealed low noxiousness, facile preparation, and eco-friendliness to be employed in polymeric membranes [19,20]. Consequently, polymer nanocomposite membranes have the potential for waste water purification towards chemicals, heavy metals, salts, microorganisms, oils, etc. In this regard, polymer/fullerene based microfiltration, nanofiltration, ultrafiltration, desalination, and reverse osmosis membranes have been developed [21,22]. The implication of polymer/fullerene nanocomposites in water treatment membranes has led to genuine innovations in the field of water purification technologies.

This review is revolutionary to portray the scientific development and advancement in the field of polymer/fullerene C_60_ nanocomposite-based water treatment membranes. Fullerene-based polymer nanocomposite membranes have recently attracted significant attention for waste water treatment and purification, mostly for the removal of toxic metals, microorganisms, chemical compounds, salts, etc. Various literature reports have been found on the design and performance of polymer/fullerene-derived ultrafiltration, nanofiltration, pervaporation, desalination, and reverse osmosis membranes. However, to the best of knowledge, such a specific review on polymer and fullerene has not seen in the literature before. The actual motive behind this review is to develop a pioneering article to portray the developments in the field of polymer/fullerene-based nanocomposite membranes. Significant literature reports on polymer/fullerene nanocomposite membranes were found between 2010 and 2022. Fewer reports developed before 2010 were mentioned in this review, so the main progress in this field during the last two decades was depicted. Thus, this state-of-the-art review highlights some auspicious zones of polymer/fullerene C_60_ membranes for water treatment. The resourcefulness of polymer/fullerene nanocomposites has accelerated membrane processes for water purification or effluents remediation. Moreover, polymer/fullerene nanocomposites have brought about numerous novelties in the field of high-performance water treatment membranes. Polymer/fullerene nanocomposite membranes have been identified as low-cost, efficient, and easily scalable materials. These membranes have been found highly permeable to water and have stable structures, selectivity, solute rejection, and low fouling. For the formation of polymer/fullerene membranes, polymers such as Nafion, polysulfone, polyamide, and polystyrene have been preferred. Some preparation methods for forming polymeric membranes include solution casting, phase inversion, electrospinning, and solid phase synthesis routes. The ensuing novel membrane materials may have capabilities to overwhelm the challenging aqueous environment for the filtration of undesirable contaminants. In other words, this article offers a groundbreaking and original review on polymer and fullerene-based membrane materials. Despite remarkable properties and the vast potential, devoted future research efforts are desirable to form high-performance nanocomposite membranes for water purification applications and to overcome the associated challenges. Future developments in the field of novel functional polymer/fullerene C_60_ nanocomposite membranes are not possible for researchers before getting prior knowledge of the reported literature in this field.

## 2. Fullerene

**Figure 1:**
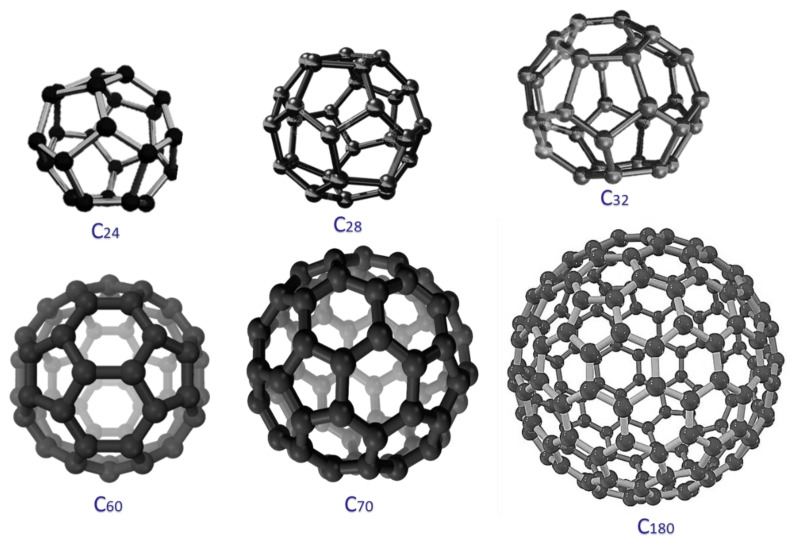
Fullerene nanostructures.

Fullerene is a symmetrical zero-dimensional nano-allotrope of carbon [23]. It is a hollow ball-like nanostructure having sp^2^ hybridized carbon atoms in its architecture [24]. The carbon atoms in fullerene form a π conjugation system [25,26]. It is made up of unique polygons, i.e., pentagons and hexagons. The cage-like fullerene nanostructure is ~1 nm [27]. Fullerene was primarily discovered in 1985 [28]. Depending upon the number of carbon atoms in the structure, the fullerene molecules can be of several types such as C_24_, C_28_, C_60_, C_70_, and C_120_ (Figure 1) [29]. Fullerene C_60_ is a commonly known fullerene molecule also referred to as buckminsterfullerene. Then, the next widely researched form of fullerene is C_70_. Fullerene molecules have revealed sole optical, electrical, magnetic, thermal, mechanical, and biomedical possessions [30,31,32]. Fullerene molecules have been primed using numerous procedures such as the microwave synthesis, arc discharge, plasma techniques, chemical vapor deposition, and numerous chemical approaches [31,32,33]. Nanocarbons such as graphene, carbon nanotube, and nanodiamond have been applied in essential technical applications including nanocomposite formation [34,35,36,37]. Nanocomposites have been formed using the in situ method, solution mixing, melt processing, spinning, printing, and other practices [38,39,40]. Fullerene molecules own exceptional physical features for nanocomposite applications [41,42,43,44]. With polymers, fullerene molecules may form an electron donor-acceptor relationship [45]. The solubility properties of fullerene in number of solvents have been explored for the nanocomposite formation [46,47,48]. The polymers such as poly(vinylpyrrolidone) have also been employed to solubilize fullerene molecules [49,50,51]. Applications areas of fullerene, functional fullerene, and nanocomposites have been observed in optoelectronics [52,53,54], solar cells [55], sensors [56], drug delivery [57], and other practical fields. In the membrane application, fullerene has been reinforced in polymeric matrices to influence final properties [58]. As compared to other nanocarbons, fullerene has a higher surface area to interact with polymers and own a lower aggregation tendency and better dispersion properties [59]. For water treatment purposes, fullerene addition may enhance homogeneity in polymeric membranes; however, appropriate surfactants and stabilizing agents need to be included to form high-performance materials [60,61]. Mostly, fullerene C_60_ molecules are reinforced in polymeric matrices to form water filtration membranes. Other fullerene forms have been rarely studied in polymeric membranes.

## 3. Polymeric Nanocomposite Membranes

**Figure 2:**
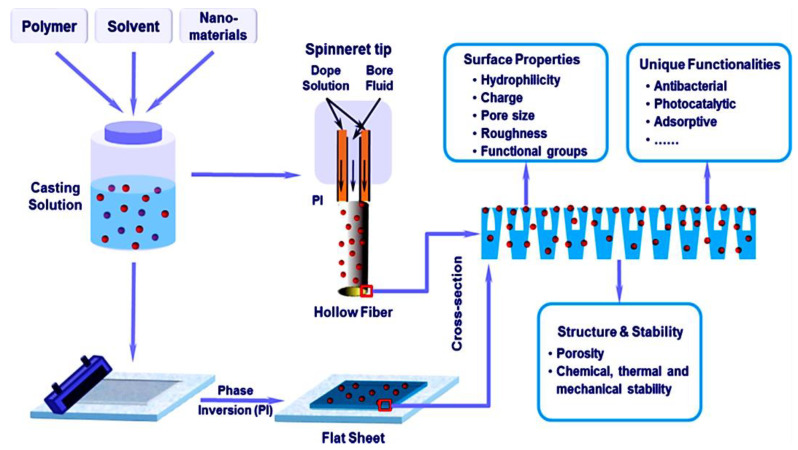
Fabrication of conventional nanocomposite membranes [130]. Reproduced with permission from Elsevier.

Membrane technologies has expanded research interest in methodological applications and technical industries [62,63,64]. Membrane technologies has been used for the remediation of environmental pollutants including noxious gases and water contaminants [65,66,67]. Relative to neat polymeric membranes, nanocomposite membranes have been beneficially applied for water treatment [68]. Membrane technologies have especially targeted domestic water management, industrial waste water treatment, and seawater desalination [69,70,71]. In the membrane filtration process, the trade-off association between the membrane permeability and the membrane selectivity is considered important [72,73,74]. Compared with neat polymeric membranes, nanocomposite membranes exhibit the advantages of superior physicochemical characteristics due to the combination of polymers and nanoparticles [75,76,77,78,79]. Moreover, the enhanced fouling resistance, optimum porosity, hydrophilicity, mechanical robustness, and heat stability have been observed [80,81,82,83,84]. Figure 2 portrays the construction of nanocomposite membranes using a widely applied phase inversion technique [85,86,87,88,89]. This scheme usually encompasses nanoparticle dispersion in a solvent and then amalgamation with a polymer solution [90,91,92]. The phase inversion technique has been used to develop microfiltration, nanofiltration, or ultrafiltration membranes [93,94,95]. Using this approach, nanocarbons such as graphene, graphene oxide, and carbon nanotubes have been reinforced in nylon [96,97,98,99,100], polysulfone [101,102,103,104,105], poly(vinyl alcohol) [106,107,108,109,110], poly(vinyl acetate) [111,112,113,114,115], etc. to develop high-performance membranes. Ammar et. al. [116] fabricated polysulfone and graphene-based membranes with the phase inversion method. The membranes were explored for the morphology and structural crystallinity [117,118,119,120,121]. Owing to electrostatic or van der Waals interactions between the matrix and the nanofiller, the water flux of the nanocomposite membranes was enhanced [122,123]. Ganesh and co-workers [124] reported polysulfone and graphene oxide-derived mixed matrix membranes. The phase inversion method was adopted as a successful method to form membranes [125,126,127,128,129].

## 4. Polymer/Fullerene Nanocomposite

**Figure 3:**
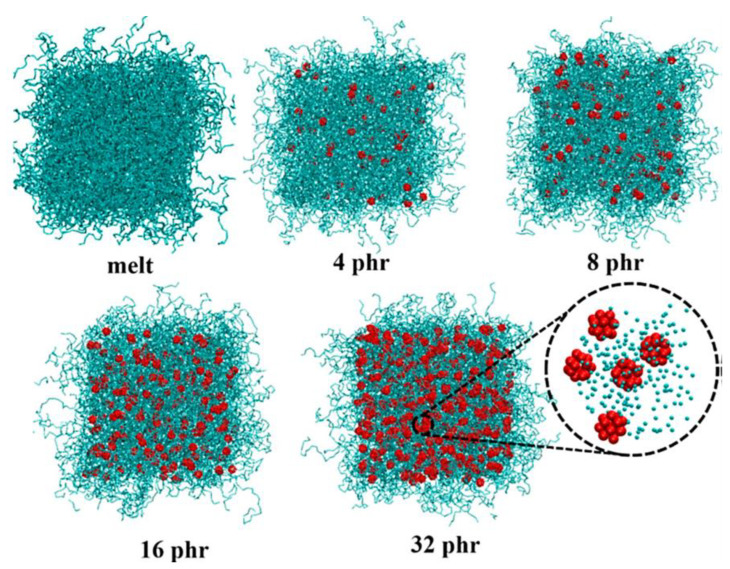
Snapshots of cis-1,4-polyisoprene and C_60_-based nanocomposite membranes with varying nanofiller contents from 0 (melt) to 32 phr [153]. Reproduced with permission Creative Commons Attribution License.

Polymer/fullerene nanocomposites have been considered for superior optical, electrical, thermal, mechanical, and processability features [131,132,133]. However, during the polymer/fullerene nanocomposite formation, agglomeration can cause major hinderance to the production of high-performance nanomaterials. The aggregation of fullerene molecules may cause poor solubility in organic solvents [134]. Consequently, the large-scale processing of polymer/fullerene nanocomposites has been studied [135]. To resolve this issue, functional fullerene molecules have been reinforced in polymeric matrices [136,137]. A range of thermoplastics, thermosets, and conjugated polymers have been used to form nanocomposites with fullerene [138,139]. Polymer/fullerene nanocomposites have been applied in wide-ranging methodological fields such as electronics [140], supercapacitors [141], and solar cells [142]. Fullerene C_60_ has been physically or covalently linked with host polymers to enhance final nanomaterial characteristics [143,144]. The systematic studies on the polymer/fullerene nanocomposites have escorted towards polymeric membranes [145]. In this regard, numerous filtration membranes have been developed using varying polymers (polyamide, polystyrene, polysulfone, etc.) and the fullerene C_60_ nanofiller [146,147,148]. Nanocomposite membranes have been focused on due to its chemical stability, mechanical resilience, rigidity, porosity, and inexpensiveness [149,150,151]. The inclusion of fullerene contents have significantly improved technological membrane parameters towards the remediation of environmental effluents [152]. Kitjanon et al. [153] developed and studied the cis-1,4-polyisoprene and C_60_-based nanocomposite membranes via coarse-grained molecular dynamics simulation. Nanoparticles with 0–32 phr fullerene were loaded, and then, simulations were performed over 200 microseconds. The photographs of the cis-1,4-polyisoprene and C_60_-based nanocomposite are given in Figure 3. In addition, the experimental results for the glass transition temperature, mechanical, and thermodynamical properties were investigated for the high-performance nanocomposites.

## 5. Implication of Polymer/Fullerene Nanocomposite Membranes in Water Treatment

**Figure 4:**
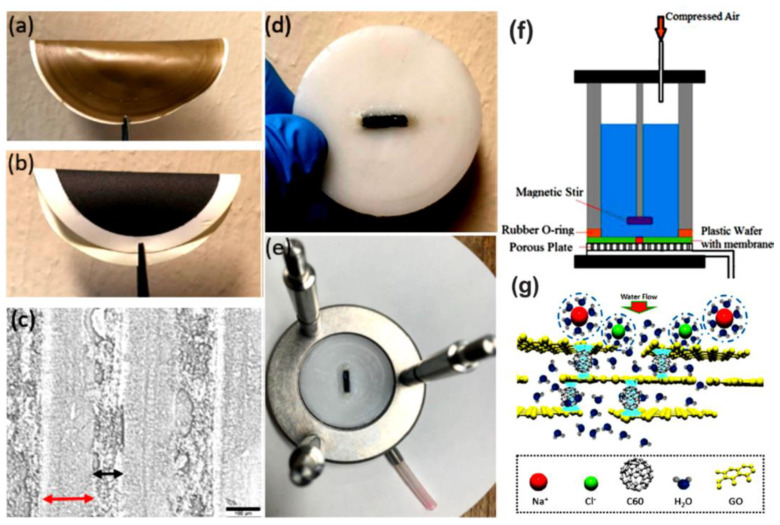
Fabrication process and water desalination setup using C_60_-grafted graphene oxide membranes. (**a**) Graphene oxide membrane without C_60_; (**b**) C_60_-grafted graphene oxide membrane; (**c**) optical micrograph of a cross-sectional area with a scale bar of 100 µm. The micrograph shows 148 µm thick graphene oxide laminates embedded in 81 µm thick epoxy; (**d**) graphene oxide-C_60_ membrane encapsulated with epoxy in a plastic disk of 47 mm; (**e**) graphene oxide-C_60_ membrane inside a water desalination setup; (**f**,**g**) schematic setup of a flat membrane made of graphene oxide and a C_60_ hybrid for water desalination [162]. Reproduced with permission from American Chemical Society.

Polymer/fullerene nanocomposite membranes have been investigated for effective salt removal from water, toxic ion separation, ion pair separation, recovery of expensive metals, and pathogenic microorganisms separation or deterioration [154]. In the sorption method, a huge range of metal ions can be recovered such as nickel, zinc, copper, cobalt, mercury, lead, arsenic, and cadmium [155,156,157]. The design of polymer/fullerene nanocomposite membranes and surface defects may influence their sorption capacities towards metals [158]. Moreover, nanocomposite membranes show better retention time [159]. Perera et al. [160] formed thin-film reverse osmosis membranes having functional hydroxy fullerene C_60_. The membranes revealed a high water flux of 26.1 L/(m^2^∙h) and the optimum salt rejection. Moreover, the hydroxy functional fullerene C_60_-derived membranes presented a high separation efficiency towards Mg^2+^/Li^+^ (separation factor: 13.1). The nanocomposite membranes were found effective for the recovery of Li^+^ ions from seawater [161]. Liu et al. [162] reported epoxy-based membranes with fullerene C_60_-grafted graphene oxide. Water desalination and ion permeation were studied through the membranes. Figure 4 displays the fabrication procedure, water desalination system, and representation of anions/cations blockage on the membrane surface. The membranes had a high water flux of up to 10.85 L/(m^2^∙h∙bar) and 0.1883 mol/(m^2^∙h∙bar) for water desalination and ion permeation, respectively. The inclusion of fullerene C_60_ revealed fine water adsorption for large water quantities. Table 1 displays the filtration performances of a few systems showing polymeric membranes with fullerene nanoparticles.

The biocompatibility of fullerene is an ultimate requirement to ensure the safety of drinking water [168]. Fullerene nanofillers embedded into various polymeric membranes influence the resulting membranes performance. The size, shape, and surface properties of fullerenes have been found important to enhance the solute selectivity, water permeability, and stability of nanocomposite membranes. Fullerene nanofillers have been used to achieve desirable pore sizes, larger surface areas, and unique surface functionalities, improving the overall membrane performance. For the fabrication of polymer/fullerene nanocomposite membranes, the surface chemistry of fullerene has been considered important to accomplish better membrane stability [169]. The presence of physical or covalent bonding of fullerene with polymers in nanocomposite membranes have been found to improve hydrophilicity and anti-bacterial, anti-fouling, and water flux performance. Moreover, interactions between fullerene and polymers also aid in the improved membrane thermomechanical stability and long-term durability during filtration and hydraulic cleaning processes. In addition, polymer/fullerene membranes possess low toxicity, long-term organic solvent stability, and enhanced membrane operating times even at higher temperatures. Molecular dynamics (MD) simulations were used to gain insights on the interactions of polymer with fullerene in membranes [170]. The interactions between a single fullerene C_60_ molecule and a membrane were evaluated by computing the free energy as a function of the distance between the C_60_ molecule and the polymer layer. The fullerene molecule was found to be absorbed into the polymer hydrophobic part with a marked increase in the stability of the membrane (~30 kcal/mol) and cause no disruption of the hydrophobic environment. According to MD studies, initial fullerene clusters need to be disaggregated to release from the membranes. Furthermore, to make the fullerene molecule soluble from the polymeric membranes, both the energetic and kinetic barriers need to be overcome. Thus, the studies revealed the kinetic stability of polymer/fullerene nanocomposite membranes, and these membranes were not found easily degradable in aqueous environments.

### 5.1. Nafion/Fullerene Nanocomposite Membranes for Water Treatment

**Figure 5:**
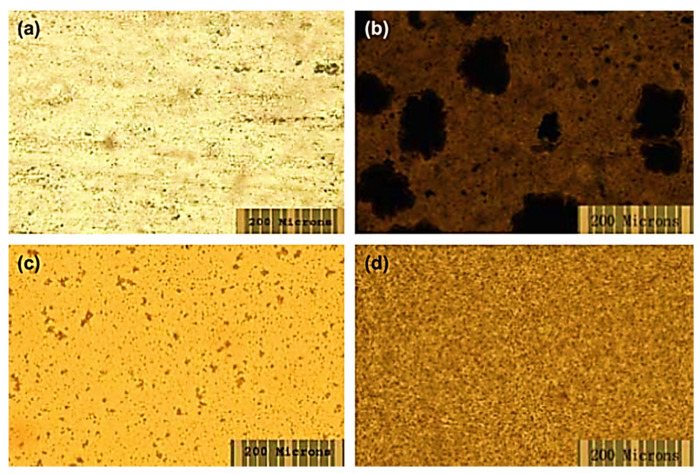
Optical micrographs of Nafion/C_60_ fullerene nanocomposites (**a**) and Nafion/polyhydroxy fullerene nanocomposites (**b**) by doping and Nafion/C_60_ fullerene nanocomposites (**c**) and Nafion/polyhydroxy fullerene nanocomposites (**d**) by solution casting [176]. Reproduced with permission from Elsevier.

**Figure 6:**
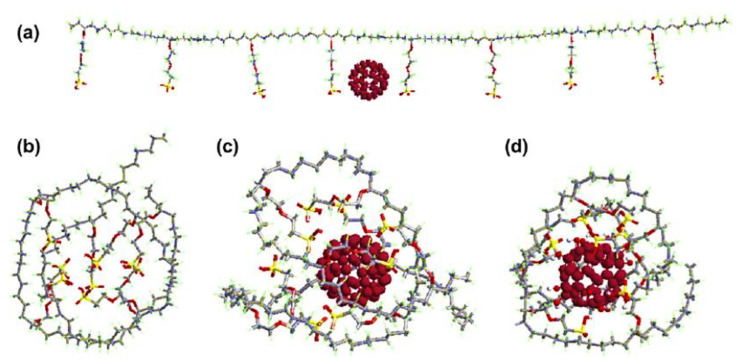
(**a**) Initial structure of C_60_ and Nafion oligomers; (**b**) snapshots of Nafion oligomers; (**c**) C_60_ and Nafion oligomers; and (**d**) polyhydroxy and Nafion oligomers (taken after 1 ns molecular dynamic simulations) [176]. Reproduced with permission from Elsevier.

Nafion is a sulfonated tetrafluoroethylene-based fluoropolymer-copolymer [171,172]. It is a widely used synthetic polymer. It has ionic properties such as ionomers. Nafion has been commonly used to form commercial membranes for energy, electronics, and environmental applications [173,174]. Nafion and fullerene-based nanocomposite membranes have been devised [175]. The use of hydroxy-modified fullerene in Nafion results in fine photoconductivity and antimicrobial characteristics. Tasaki et al. [176] fabricated a Nafion/fullerene C_60_ nanocomposite with the solution casting technique. The neat fullerene C_60_ and the polyhydroxy fullerene were used as nanofillers. Figure 5 demonstrates the optical micrographs of the solution-cast nanocomposite membranes. Two types of membranes were prepared, i.e., through the doping process and by solution casting. In the doping process, toluene was used as a solvent. On the other hand, dimethyl acetamide was used as a solvent in the solution route. In the doping route, functional fullerene nanoparticles formed large aggregates. In the case of the solution route, finely dispersed fullerene and functional fullerene nanoparticles can be observed. Thus, the solution route was found ideal due to better miscibility properties between the Nafion matrix and the nanofiller. Figure 6 expresses the molecular dynamic simulations of the Nafion/fullerene nanocomposite membranes. The fullerene molecule can be seen totally wrapped by the Nafion oligomer, screening better mutual interactions. The water uptake of the membranes was measured through soaking in water (wet condition) and under a 25% RH (dry condition). The 1 wt % Nafion/C_60_ and 3 wt % Nafion/C_60_ membranes were tested. Both the Nafion/C_60_ nanocomposite membranes were found to hold more water than the neat Nafion, under a 25% RH. Consequently, the higher wet and dry water uptakes of nanocomposite membranes were observed, compared with those of the neat Nafion membrane. The water molecules were suggested to be trapped in the interfaces between the C_60_ aggregates and Nafion domains, depending upon the morphological form of the nafion/C_60_ nanocomposite. In the Nafion/fullerene nanofiltration membranes, the internal porosity and permeability were found to be affected by the packing of the polymer chains and dispersion of the fullerene nanoparticles [175]. The presence of fullerene was supposed to interrupt the packing manner of the polymer chains and interfacial morphology to generate nanopores in the Nafion/fullerene nanofiltration membranes for ion separation purposes. Nevertheless, few Nafion/fullerene systems have been studied up till now for water treatment, so further research efforts are found desirable in this category.

### 5.2. Polysulfone/Fullerene Nanocomposite Membranes in Water Treatment

Polysulfone is a commercial thermoplastic polymer [177]. Polysulfone own fine properties of chemical, thermal, and mechanical stability. Owing to unique processability and physical features, polysulfone has found wide-ranging applications in membranes, coating, nanocomposites, and other technical fields [178]. Penkova et al. [179] fabricated mixed-matrix membranes of polysulfone and fullerene C_60_ (contents up to 5 wt %). The nanocomposite membrane with a 5 wt % fullerene content revealed fine transport properties towards the pervaporation of an ethyl acetate-water mixture. The sorption and contact angle measurement data for the membranes are given in Table 2. The inclusion of nanofillers enhanced the sorption characteristics. Moreover, the hydrophilic features of the membrane surface were improved with the fullerene addition. The pervaporation mechanism can be explained through the solution-diffusion process [180]. Especially, the permeability is directly related to the solubility and diffusivity of the solute molecules. In this way, the mass transfer of analyte and water through the membranes can be analyzed. However, limited polysulfone/fullerene systems have been studied so far, and thorough research efforts needed to form high-performance membranes in this category.

### 5.3. Polyamide/Fullerene Nanocomposite Membranes towards Water Treatment

**Figure 7:**
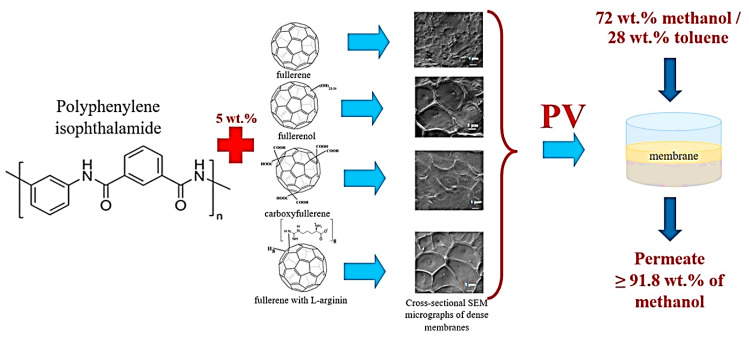
Graphical development of novel polyphenylene isophthalamide pervaporation (PV) membranes modified with various types of fullerene C_60_ derivatives [166]. Reproduced with permission from Elsevier.

**Figure 8:**
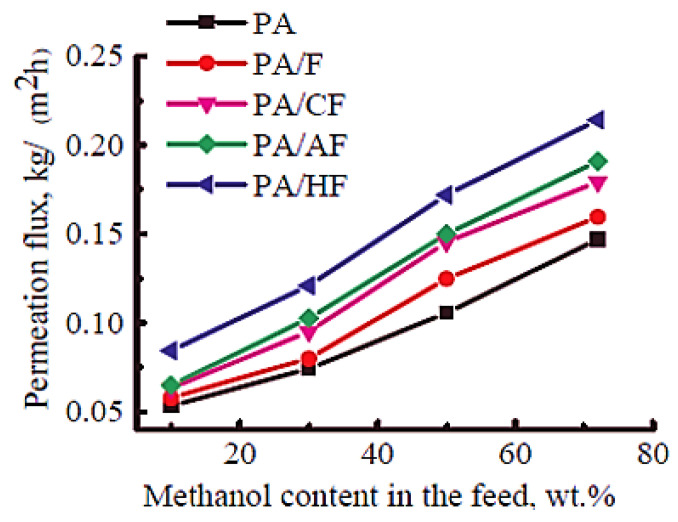
Dependence of the permeation flux on the methanol content in feed for membranes based on neat PA and nanocomposites with fullerene and fullerene derivatives during pervaporation of methanol-toluene mixtures with 10–72 wt % methanol (22 °C) [166]. PA, polyphenylene isophthalamide; PA/F, polyphenylene isophthalamide/fullerene; PA/CF, polyphenylene isophthalamide/carboxyfullerene; PA/AF, polyphenylene isophthalamide/fullerene derivative with L-arginine; PA/HF, polyphenylene isophthalamide/polyhydroxylated fullerene. Reproduced with permission from Elsevier.

Polyamide is a thermoplastic polymer having repeating amide bonds in the backbone. Polyamides occur naturally and can be synthesized using diamine and dicarboxylic acid monomers [181]. Polyamide has been effectively used in membrane applications [182]. Plisko et al. [167] designed a polyamide and hydroxy functional fullerene C_60_-based thin-film nanocomposite membrane for water treatment. It has been observed that the inclusion of a 5 wt % nanofiller promoted antifouling features and organic matter elimination properties of the membranes. Dmitrenko et al. [166] adopted a polyamide, i.e., polyphenylene isophthalamide filled with 5 wt % fullerene derivatives (carboxyfullerene, polyhydroxylated fullerene and fullerene derivative with L-arginine). In this regard, various pervaporation membranes have been designed as polyphenylene isophthalamide/fullerene (PA/F), polyphenylene isophthalamide/carboxyfullerene (PA/CF), polyphenylene isophthalamide/fullerene derivative with L-arginine (PA/AF), and polyphenylene isophthalamide/polyhydroxylated fullerene (PA/HF). The solid phase synthesis route was used to fabricate mixed matrix pervaporation membranes (Figure 7). The transport properties were studied for the azeotropic methanol-toluene (72/28 wt %) mixture. Figure 8 depicts the permeation flux for the mixed matrix pervaporation, which was increased with the increasing methanol content in feed. Moreover, the inclusion of fullerene derivative to the polymeric membranes enhanced the permeation flux in order of PA < PA/F < PA/CF < PA/AF < PA/HF. Relative to the neat pervaporation membrane, the permeation flux of the nanocomposite membrane was increased by 1.6 times, due to the effect of the inclusion of fullerene nanoparticles. Furthermore, the nanocomposite membranes have enhanced the surface roughness and the surface hydrophilicity properties, relative to the neat polyphenylene isophthalamide membrane. The polyhydroxylated fullerene-based membrane revealed superior performance compared with the other nanocomposite membranes. Consequently, the PA/HF membrane with a 5 wt % nanofiller content increased the permeation flux to 0.084–0.214 kg/(m^2^∙h) and resulted in the selectivity of 95.9 wt % for methanol. The results were attributed to the affinity of the polyhydroxy functionality of the fullerene molecules permeating methanol. The results were also suggestive of the nanocomposite membrane pore size modification with the addition of altered fullerene derivatives [183]. Comprehensive efforts are still desirable to explore the interaction of the modified fullerene nanoparticles with the polyamide chains actually operating to promote the membrane permeation and selectivity.

### 5.4. Polystyrene/Fullerene Nanocomposite Membranes for Water Treatment

**Figure 9:**
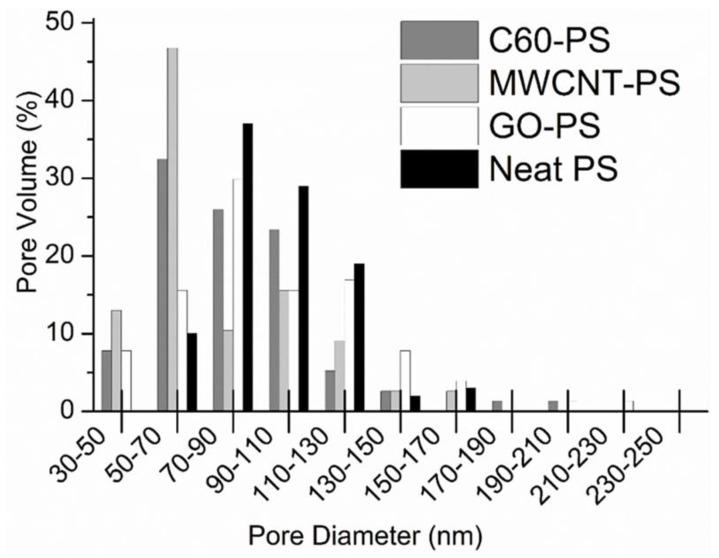
Distribution of the pore volume measured from scanning electron microscopy of nanofibers [188]. PS, polystyrene; C_60_-PS, fullerene-polystyrene; MWCNT-PS, multi-walled carbon nanotube-polystyrene; GO-PS, graphene oxide-polystyrene. Reproduced with permission from Elsevier.

Polystyrene is a widely used commercial thermoplastic polymer [184,185]. Polystyrene is usually made from styrene monomer. It is an inexpensive, light-weight, clear, hard, and brittle polymer. Polystyrene has been frequently used to form nanocomposites. Alekseeva et al. [163] formed fullerene C_60_-filled polystyrene nanocomposite. The ultrafiltration membranes were designed using the phase inversion technique. The static protein sorption tests were carried out for the polystyrene/C_60_ nanocomposite membranes. With the fullerene nanoparticle loading, protein sorption was reduced along with the flux reduced recovery rates. The result was observed due to better fullerene dispersion and barrier effects in the membranes. Moreover, the Langmuir model was used to study the Cu^2+^ ions removal efficiency. It was found that the fullerene addition enhanced the membrane affinity towards Cu^2+^ ions due to better interactions. The fullerene C_60_ has also been incorporated in polystyrene blend matrices to form water treatment membranes [186,187]. von Reitzenstein et al. [188] performed a comparative study on neat polystyrene, polystyrene/fullerene C_60_, polystyrene/graphene oxide, and polystyrene/multi-walled carbon nanotubes. The electrospinning technique was used to form nanocomposite nanofibers. Electrospun nanofibers were then used to form nanofiltration membranes. The inclusion of nanofillers in the electrospun polystyrene nanofibers slightly enhanced the diameter, reduced the bead formation and caused the homogeneous surface pore size distribution. Figure 9 displays the surface pore volume distribution of the nanofiber. The mean pore diameters of the neat polymer and polystyrene/graphene oxide was found similar, which were 70–90 nm.

**Figure 10:**
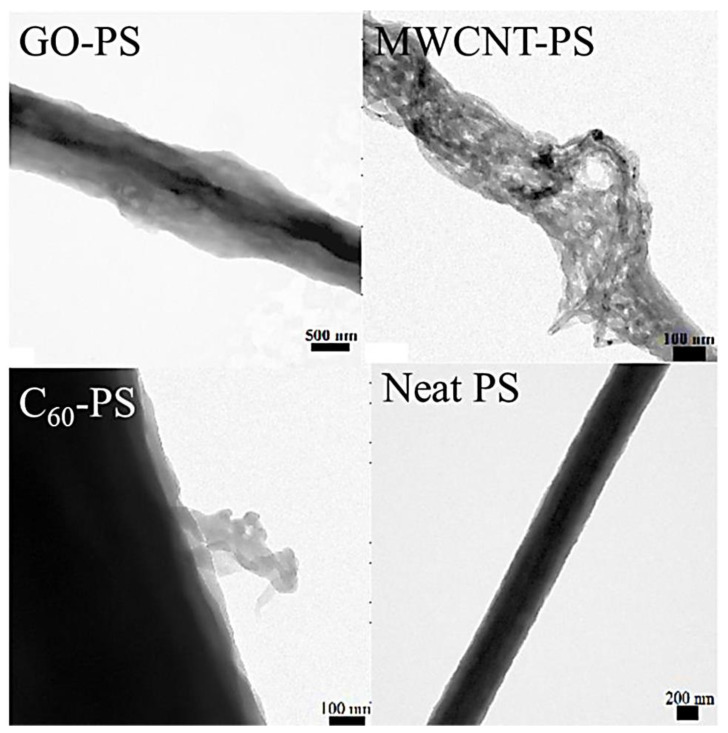
Transmission electron microscopic images of the neat PS and the nanocomposite nanofiber [188]. PS, polystyrene; C_60_-PS, fullerene-polystyrene; MWCNT-PS, multi-walled carbon nanotube-polystyrene; GO-PS, graphene oxide-polystyrene. Reproduced with permission from Elsevier.

On the other hand, the polystyrene/fullerene C_60_ and the polystyrene/multi-walled carbon nanotube had a reduced mean pore diameter of 50–70 nm. The effect was observed probably due to more stability and less pore formation tendency of fullerene and nanotube-filled nanocomposites. The transmission electron microscopy (TEM) was used to inspect the morphologies of the neat polystyrene nanofiber and the nanocarbon-dispersed nanocomposite nanofiber (Figure 10). The polystyrene/graphene oxide-based nanofiber showed a flaky appearance due to the presence of nanosheets. The nanotube can be observed as tangled threads in the polystyrene/multi-walled carbon nanotube nanocomposites. The polystyrene/fullerene nanofibers were opaque and dense, although few fullerene aggregates can be identified as flaky edges on the fiber surface. The polymer/nanocarbon nanofibers for the filtration membranes must be further investigated for the mechanism of pore creation, pore formation frequency control, and precise nanofiber dimensions [189].

## 6. Challenges, Future, and Summary

There is a mammoth necessity of better-quality approaches to the use of fullerenes and derived nanomaterials in high tech industries [190]. Most prominently, fullerene-derived nanomaterials have been absorbed for membranes or coatings [191], electronics [192], energy devices [193], biomedical areas [194], and other fields. The future implication of fullerene-based nanomaterials has been suggested for the aerospace or automotive, civil engineering, and other high-performance applications. In membrane applications, polymer/fullerene nanocomposites have been applied due to remarkable morphological, mechanical, barrier, electrical, and other physical properties [195]. In polymer/fullerene nanocomposite membranes, nanofiller dispersal and matrix-nanofiller interactions have been found desirable to develop high-performance nanostructures. Accordingly, the fullerene dispersion has been identified as an important challenge of these membranes. In this regard, modified fullerene derivatives need to be employed. Moreover, precise controls over the pore size, pore structure, microstructure, membrane surface roughness, membrane wettability, etc. (defining the membrane performance parameters) remain as major encounters [196,197]. Furthermore, comprehensive studies are still desired to explore the mechanism of membrane separation processes. Solution-diffusion processes leading to water filtration need to be explored experimentally and theoretically, to profoundly identify the separation mechanism. Another important challenge is the discovery of additional all-inclusive categories of polymer/fullerene membranes beyond Nafion/fullerene, polyamide/fullerene, polysulfone/fullerene, and polystyrene/fullerene nanocomposite membranes known so far towards water treatment. The above-mentioned challenges can be overcome by using modified fullerene nanoparticles to attain promising forthcoming membrane nanomaterials. For future membrane utility, the self-assembly of fullerene molecules and network formation with polymeric matrices must be considered [198,199,200].

Consequently, polymer/fullerene nanocomposites capture a special position as separation membranes in the water treatment industry. These membranes have attained increasing research attention due to performance advantages, low operating expenses, high selectivity, and easy scale-up. In fact, the primary function of fullerene has been found as physical/chemical binders within the polymeric chains of nanocomposite membranes for optimizing essential mechanical properties, structural stability, thermal stability, and membrane properties. Certainly, inclusion of fullerene in the matrix creates van der Waals forces and/or covalent interactions with the polymer chains, leading to reinforcement and superior physical features. Moreover, the variations in fullerene loading levels cause dominant effects on the reinforcement and membrane properties. Subsequently, polymer/fullerene nanocomposites have been found as fast emerging separation membranes for clean water resources. Among all available polymer/nanocarbon membranes, polymer/fullerene nanocomposites have been considered as the energy-efficient and green technology for separation of impurities from aqueous solutions due to optimum nanoporous membrane features. Recently, polymer/fullerene nanocomposite membranes have been considered significant for resolving technical and commercial challenges towards separation and purification technologies. The development of distinct polymer/fullerene nanostructured membranes with exclusive properties not only solves the trade-off issue related to water treatment technologies, but also opens new pathways towards real-time applications. The newly fabricated polymer/fullerene membranes have been found superior in terms of selectivity, permeability, and long-term stability. Thus, the research on these novel nanocomposite membranes may continue to develop better cost-effective water treatment systems to decrease the overall capital investment.

In short, this cutting-edge overview grants an analysis on the use of polymer/fullerene C_60_ nanocomposite membranes for water remediation. Fullerene and modified fullerene nanofillers have been incorporated in polymers to develop nanocomposite membranes. The enhanced polymer/fullerene nanocomposite properties lead to several prospects to revolutionize the related potential for waste water purification. Plentiful research efforts have been fixated on refining the polymer/fullerene nanocomposite membrane properties such as water permeability, salt rejection, ion separation, overall separation efficiency, antifouling performance, and other membrane topographies. Moreover, fullerene-based nanofillers improve the membrane surface properties, have low cost, and enhance the long-term stability of the membranes. To widen the use of polymer/fullerene in membranes, devotion must be taken to augment polymer-nanocarbon interactions, nanofiller dispersibility, membrane stability, membrane permeability, separation competence, and practicable membrane parameters to solve current glitches in this field.

## Figures and Tables

**Table 1:** Specifications of a few polymeric membranes with fullerene C_60_ nanofillers for water purification.

Nanofiller	Size of Nanofillers (nm)	Pore Size of the Membrane	Filtration (L/m^2^∙h∙bar)/(LMH∙bar)	Ref.
C_60_	0.375 (radius)	-	-	[163]
C_60_	-	C_60_ addition caused a bigger pore size.	-	[164]
C_60_	-	17 nm	-	[150]
C_60_	0.375 (radius)	-	-	[165]
C_60_	4.4–122	-	-	[154]
C_60_	9–15	Small pores 5 wt % fullerenol	0.084–0.214 kg/(m^2^∙h)	[166]
Polyhydroxylated C_60_	-	0.639 nm	6.7 LMH∙bar	[161]
Functional C_60_	~1	0.86 to 0.59 nm	26.1 LMH	[160]
C_60_	14–59	33–34 nm to 53–55 nm	-	[167]

**Table 2:** Sorption characteristics and contact angles of polysulfone and polysulfone/fullerene C_60_ dense membranes [179]. Reproduced with permission from Springer.

Membrane	Sorption (%)		Contact Angle (°)
	Ethyl Acetate:Water Ratio (2%:98%)	Ethyl Acetate:Water Ratio (4%:96%)	
Polysulfone	0.8	8.4	62
Polysulfone with 3 wt % C_60_	1.3	9.7	66
Polysulfone with 5 wt % C_60_	3.2	11.7	79
